# Prevalence of common autosomal recessive mutation carriers in women in the Southern Vietnam following the application of expanded carrier screening

**DOI:** 10.1038/s41598-024-57513-0

**Published:** 2024-03-29

**Authors:** Xuan-Hong To-Mai, Huu-Trung Nguyen, Thanh-Truc Nguyen-Thi, Thuy-Vy Nguyen, My-Nuong Nguyen-Thi, Ke-Quan Thai, Minh-Thi Lai, Tuan-Anh Nguyen

**Affiliations:** 1 University of Alberta, Edmonton, Canada; 2 University of Nam Can Tho, Can Tho, Vietnam; 3 University of Medicine and Pharmacy at Ho Chi Minh City, Ho Chi Minh City, Vietnam; 4 University Medical Center–Branch 2, Ho Chi Minh City, Vietnam; 5 University of Science, Vietnam National University Ho Chi Minh, Ho Chi Minh City, Vietnam; 6 Ktest Company, Ho Chi Minh City, Vietnam; 7 Saigon University, Ho Chi Minh City, Vietnam; 8 Molecular Biomedical Center, University Medical Center, Ho Chí Minh City, Vietnam

**Keywords:** Autosomal recessive disorders, Carrier screening, Expanded carrier screening, Gene mutation, Vietnamese-specific carrier screening panel, X-linked disorders, Genetics, Medical research

## Abstract

The common autosomal recessive (AR) mutation carrier is still unknown in Vietnam. This study aims to identify the most common AR gene mutation carriers in women of reproductive age to build a Vietnamese-specific carrier screening panel for AR and X-linked disorders in the preconception and prenatal healthcare program. A cross-sectional study was conducted at University Medical Center–Branch 2 in Ho Chi Minh City from December 1st, 2020, to June 30th, 2023. 338 women have consented to take a 5 mL blood test to identify 540 recessive genes. The carrier screening panel was designed based on the American College of Medical Genetics and Genomics (ACMG)-recommended genes and suggestions from 104 clinical experts in Vietnam. Obstetricians and genetic experts counseled all positive testing results to discuss the possibility of recessive diseases in their offspring. The most common recessive disorders were defined at a prevalence of 1 in 60 or greater, and those were added to a Vietnamese-specific carrier screening panel. 338 non-pregnant and pregnant women underwent the expanded carrier screening (ECS). The carrier frequency was 63.6%, in which 215 women carried at least one AR gene mutation. *GJB2* hearing impairment was identified as the most common chronic condition (1 in 5). The second most common AR disorder was beta-thalassemia (1 in 16), followed by cystic fibrosis (1 in 23), G6PD deficiency (1 in 28), Wilson’s disease (1 in 31), Usher’s syndrome (1 in 31), and glycogen storage disease (1 in 56). Seven common recessive genes were added in ethnic-based carrier screening. Women in the South of Vietnam have been carried for many recessive conditions at high frequency, such as hearing impairment, genetic anemia, and cystic fibrosis. It is necessary to implement a preconception and prenatal screening program by using seven widely popular AR genes in a Vietnamese-specific carrier screening panel to reduce the burden related to AR and X-linked disorders.

## Introduction

**Figure 1:**
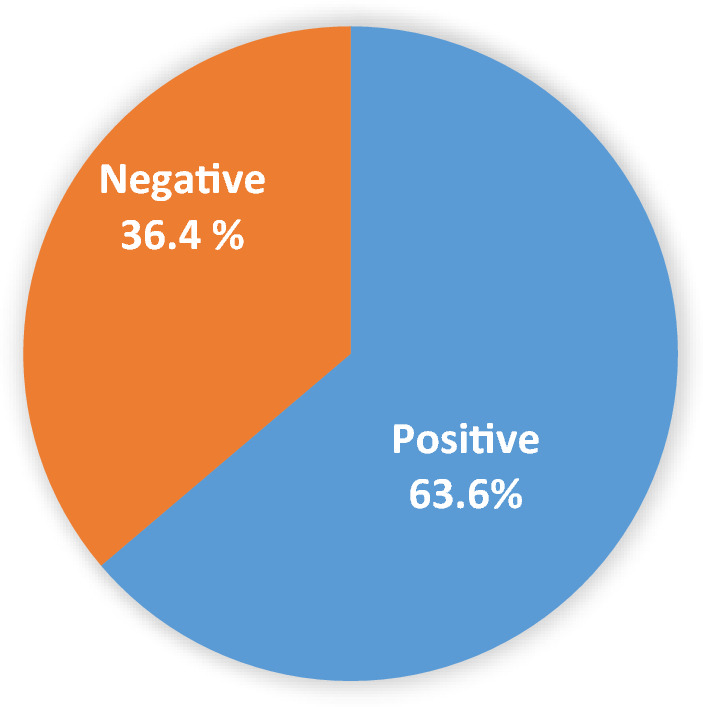
The overall prevalence of carriers for at least one recessive disease.

Carrier screening for autosomal recessive (AR) and X-linked disorders significantly benefits the reproductive healthcare program^1^. Generally, AR carriers aren’t aware of their carrier status due to a lack of clinical symptoms and only just realized after their child is diagnosed with a recessive condition^2^. That is why the AR carrier screening program has played a crucial role in building up healthy offspring generation regarding preventable healthcare and diagnosis of early-onset disorders^3^. In the literature, there are two policies of carrier screening, including the expanded carrier screening (ECS), also known as panel-based screening and target carrier screening. Both screening strategies have their benefits and disadvantages. A target carrier screening helps to identify more recessive conditions in high-risk populations at a low cost. At the same time, an ECS increases the detection rate of the condition, even in minority groups, without causing stigmatization in the community^1^. According to Rowe et al., the more AR gene mutations are screened, the more carriers will be detected^1^. However, ECSs are highly expensive and usually give out unrealistic possibilities when implemented in low carrier frequencies. Therefore, knowing the common recessive gene panel becomes an essential foundation for setting up a specific carrier screening program in the population (Fig. 1).

To our knowledge, ECS was recommended by specialists in the European Society of Human Genetics (ESHG) in 2016 and the American College of Obstetricians and Gynecology (ACOG) in 2017 with specific guidelines that demonstrated the feasibility of applying for pre-pregnancy and prenatal screening, capacity to link with newborn screening program and establishment of a suitable gene panel based on clinical evidence of severe childhood-onset diseases with the frequency of 1 in 100 or greater^4^. In terms of clinical practices, each country has designed specific panel screening based on ethnicity, popular AR disorders, and acceptability of individuals. In the UK, a preconception and prenatal carrier screening panel has been established since 2017 to early diagnose genetic anemias (beta-thalassemia, sickle cell anemia), Tay-Sachs disease, and cystic fibrosis^5^. In China, Shi et al. applied in 2021 an ECS panel with 11 conditions, including dystrophinopathies (Duchenne muscular dystrophy and Becker muscular dystrophy), anemias (alpha-thalassemia and beta-thalassemia), autosomal recessive deafness (DFNB4), phenylketonuria (PKU), glycogen storage disease type II, hemophilia A and B, and spinal muscular atrophy (SMA)^6^. His study recruited 1321 Chinese Hong Kong women who underwent the ECS and reported the carrier frequency at 19.23%^6^. Fu et al. also reported in China that 66.9% of participants preferred preconception recessive carrier screening for deafness^7^. In Australia, Beard et al. conducted an ECS in 10 women (8 pregnant women and 2 women of reproductive age) for cystic fibrosis (CF), spinal muscular atrophy (SMA), and fragile X syndrome (FXS). The results showed that all of them supported this screening positively and suggested doing this test before preconception^8^. In the Netherlands, Mathijssen et al. found that 97% of participants who underwent a preconception and antenatal carrier screening test for 4 recessive disorders (PCH2, FADS, rhizomelic chondrodysplasia punctata type 1, and osteogenesis imperfecta) wanted to recommend it to others^9^. Those studies have proved the necessity and feasibility of designing an ethnic-specific-based carrier screening panel to screen AR and X-linked diseases.

In Vietnam, the public perception of AR carriers is still unaware in reproductive healthcare due to a lack of recessive condition data. Although AR and X-linked disorders have led to up to 20% of neonatal mortality and accounted for 18% of infant hospitalization due to chronic disability (Ministry of Health, Vietnam, 2018, http://www.moh.gov.vn), an official Vietnamese-specific carrier screening panel in Vietnam has not been established yet. The newborn screening program for Glucose-6-phosphate dehydrogenase (G6PD) deficiency and congenital hypothyroidism has been implemented in several maternity hospitals but hasn’t significantly impacted public health. Whereas the prenatal screening program for inherited anemia, especially beta-thalassemia, which has been applied since 2013^10^, showed a high prevalence of thalassemia carriers in Vietnamese pregnant women (6.4% for alpha-thalassemia and 1.32% for beta-thalassemia). This early diagnosis allows their children to gain more opportunities to have premature and effective treatment (bone marrow transplantation). Recently, Tran et al. conducted a cohort study in 985 Vietnamese women and men to evaluate autosomal dominant and recessive mutation genes in the ECS panel with 118 AR conditions. The results showed 7 popular recessive gene mutations, including *GJB2* (deafness), *VPS13B* (Cohen syndrome), *HBB* (beta-thalassemia), *SLC25A13* (Citrin deficiency), *GCNT2* (cataract 13), *TMEM67* (Joubert syndrome), *PAH* (Phenylketonuria)^11^. Facing a very high AR carrier prevalence in Vietnam, the authors suggested a larger panel-based has been recommended to build up an optimal ECS in the Vietnamese population^11^.

Responding to the urgent need to improve the quality of reproductive healthcare programs, including preconception and prenatal counseling for AR and X-linked disorders in Vietnam, we conducted this study to identify the prevalence of most common recessive conditions in all non-pregnant and pregnant women coming to University Medical Center–Branch 2 in Ho Chi Minh city. Following the results, a comprehensive and specific carrier screening panel will be designed and implemented in Vietnam's preconception and prenatal care programs.

## Results

338 Vietnamese women who accepted ECS were recruited at University Medical Center–Branch 2 in Ho Chi Minh City from December 1st, 2020, to June 30th, 2023. The mean age of participants was 27.3 ± 6.5 years. 25 of them were pregnant with gestational age at 12^+3^ weeks of gestation. Most (73%) live in urban areas in the South of Vietnam. The family genetic histories had shown unremarkable notice in these women.

**Figure 2:**
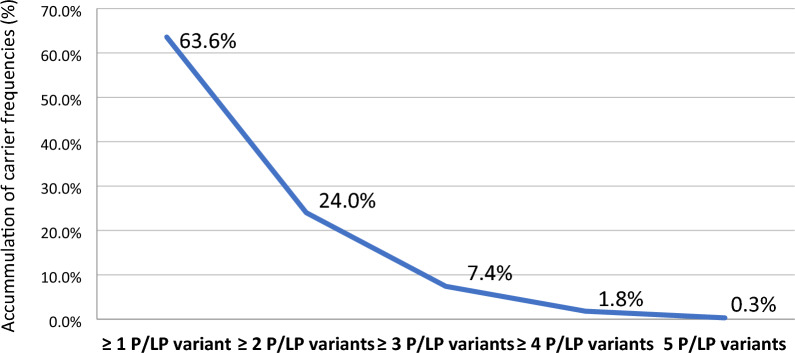
Carrier frequencies of one or more recessive disorders with pathogenic (P) and likely pathogenic (LP) variants in 540 genes of 338 Vietnamese women.

540 genes for AR and X-linked disorders were analyzed in 338 non-pregnant and pregnant women. The carrier prevalence for at least one recessive disease was 63.6% (215/338), and detailed carrier frequencies of recessive conditions are presented in Table 1. Among 215 women carriers, 134 individuals (63.6%) were carried for one recessive disease, and one woman (0.3%) had as many as 5 recessive diseases (Fig. 2). Only one pregnant woman carried 2 recessive genes (*GJB2* and *ATP7B*, both were found in heterozygous and pathogenic type) at 7^+1^ weeks, and 11 of pregnant women carried only one recessive gene (*GJB2, G6PD, CFTR*). All pregnant women carrying recessive genes followed the special prenatal care for high-risk pregnancies.

**Table 1:** Top 12 recessive disorders with the highest carrier rate (at prevalence over 1 in 100) of AR and X-liked disorder in the study population.

Gene	AR chronic conditions	Number of cases identified	Carrier frequency (%)	Carrier frequency (1 in)
*GJB2*	GJB2-related hearing impairment	67	19.8	1 in 5
*HBB*	Beta-thalassemia	21	6.2	1 in 16
*CFTR*	Cystic fibrosis	15	4.4	1 in 23
*G6PD*	G6PD deficiency	12	3.6	1 in 28
*ATP7B*	Wilson disease	11	3.3	1 in 31
*USHA*	Usher syndrome	11	3.3	1 in 31
*GAA*	Glycogen storage disease	6	1.8	1 in 56
*AGXT*	Hyperoxaluria	5	1.5	1 in 68
*GALC*	Glucose metabolic disorder	5	1.5	1 in 68
*GRHPR*	Hyperoxaluria primary, type II	4	1.2	1 in 85
*RDH12*	Leber congenital amaurosis 13	4	1.2	1 in 85
*RPGRIP1L*	Meckel syndrome, type 5	4	1.2	1 in 85

For female carriers who were married, their partners were invited to perform ECS. Unfortunately, only one male accepted this test and got a negative result.

Table 2 illustrates the detailed characteristics of the twelve top gene carriers. The most common gene carrier detected was *GJB2* (19.8%), in which 98.4% of them were pathogenic type, and c.109G>A (p.Val37Ile) variant was found significantly dominant (85.1%, 57/67). Other variants included c.299_300del (p.His100Argfs*14), c.456C>G (p.Tyr152*), c.427C>T (p.Arg143Trp) and c.235delC (p.Leu79Cysfs*3) showing the small rate around 1.5%.

**Table 2:** Characteristics of twelve top carrier rates described popular variant in 338 Vietnamese women.

Gene	Associated disorder	mRNA accession	Variant type	Nucleotide change	Protein change	dbSNP	No of cases	Allele frequency	gnomAD_EAS	ACMG criteria
*GJB2*	Hearing impairment	NM_004004.6	Missense	c.109G>A	p.Val37Ile	rs72474224	57	0.1805	0.0827	PS3 PS4 PM1 PM5 PP1 PP3
*HBB*	Beta-thalassemia	NM_000518.5	Missense	c.79G>A	p.Glu27Lys	rs33950507	18	0.0533	0.0013	PS3 PS4 PM1 PM5
*CFTR*	Cystic fibrosis	NM_000492.4	INTRON	c.1210-11T>G	p.?	rs73715573	12	0.0355	0.0202	PS3 PS4 PP5
*G6PD*	G6PD deficient	NM_000402.4	Missense	c.961G>A	p.Val321Met	rs137852327	5	0.0148	0.0039	PS3 PS4 PP2 PP3
*ATP7B*	Wilson disease	NM_000053.4	Missense	c.2549C>T	p.Thr850Ile	rs777629392	4	0.0059	0.000111	PS4 PM2 PP3 PP5
*USH2A*	Usher syndrome, type 2A	NM_206933.4	Missense	c.6929C>T	p.Thr2310Met	rs151057466	3	0.0089	0.0003	PS4 PM2 PP3
*GAA*	Glycogen storage disease II	NM_000152.5	Missense	c.752C>T	p.Ser251Leu	rs200856561	3	0.0059	0.00193	PS3 PS4
*AGXT*	Hyperoxaluria, primary, type 1	NM_000030.3	Missense	c.32C>G	p.Pro11Arg	rs34116584	5	0.0148	0.0015	PS3 PS4-M PP3
*GALC*	Glucose metabolic disorder	NM_000153.4	Missense	c.1901 T>C	p.Leu634Ser	rs138577661	2	0.0059	0.0087	PS3 PP1 PP3 PP5
*GRHPR*	Hyperoxaluria, primary, type II	NM_012203.2	Frame-shift	c.864_865delTG	p.Val289fs*22	rs180177321	4	0.0118	0.0009	PVS1 PS4-M PP5
*RDH12*	Leber Congenital Amaurosis 13	NM_152443.3	Missense	c.164C>T	p.Thr55Met	rs766631462	3	0.0089	0.0001	PS3 PS4-M PM2 PM5 PP3
*RPGRIP1L*	Meckel syndrome type 5	NM_015272.5	Frame-shift	c.3299_3300dupTC	p.Ala1101fs*34	rs797045104	2	0.0059	0.00177	PVS1 PP5-M

Four non-pregnant women carried *GJB2* with c.109G>A (p.Val37Ile) variant found in the pathogenic homozygous type. These four women were of reproductive age from 22 to 34 years old. One woman has married with gravida 1 and parity 1. Her son is 4 years old and healthy. Her husband was accepted to be tested and presented negative for *GJB2* mutation. Three remaining women were single. All four women declared that they had no health issues before performing the ECS test and weren’t aware of hearing impairment. After receiving the positive test, they underwent an auditory check and were confirmed a mild hearing deficiency by an otolaryngologist. Three single women received a post-test consultation and understood that it is required to have the partner’s *GJB2* test to evaluate the risk of hearing impairment in their future babies.

The second common recessive gene was *HBB* (6.2%), mainly in heterozygous and pathogenic types. The c.79G>A (p.Glu27Lys) variant was the popular variant (85.7%, 18/21). No pregnant women carrying the *HBB* gene were found in the present study.

*CFTR* was identified as the third common recessive carrier in our study at 4.4%, in which c.1210-11T>G found 80% (12/15) in heterozygous and pathogenic type. Two pregnant women were detected carrying *CFTR* at 7^+0^ and 7^+4^ weeks of gestation. Those pregnancies were followed at the high-risk pregnancy department until giving birth.

The fourth prevalence of recessive condition was G6PD deficiency coded by the *G6PD* gene with the carrier frequency 3.6% and c.961G>A (p.Val321Met) often observed in heterozygous and pathogenic type (42.7%, 5/12).

The fifth and sixth most common AR conditions were Wilson’s disease and Usher syndrome, determined by *ATP7B* and *USH2A*. Its carrier frequency was the same rate of 3.3%. The *ATP7B* c.2459C>T (p.Thr850Ile) variants were defined as likely to be the possible pathogenic type (45.5%, 5/11). No pregnant woman was detected carrying *ATP7B* in this study. For the *USH2A* variant, c.6969C>T (p.Thr2310Met) was a popular likely pathogenic type (27.3%, 3/11) illustrated in Table 2.

The seventh most popular recessive gene was *GAA*, with a prevalence of 1.8%. The dominant variant was c.761C>T (p.Ser254Leu) in heterozygous and pathogenic type (40%, 2/5) shown in Table 2.

The rest of the common AR conditions were detected at 1.2% and 1.5%. Those chronic disorders were related to metabolic disorders and congenital anomalies coded by *AGTX, GALC, GRHPR, RDH12, and RPGRIP1L* (Table 2).

## Discussion

### The prevalence of AR carrier in Vietnam

Analyzing 540 genes based on ACMG-recommended genes and 104 Vietnamese experts’ suggestions in 338 Vietnamese women, we found the carrier prevalence for at least one AR or X-linked condition was 63.6% (Fig. 1). This result was distinguished from previous studies due to differences in popular demographics, sample size, and the applied ECS gene panel. In the USA, Punj et al. identified 78% of carriers with at least one positive variant after performing an ECS testing in 728 genes for medically actionable conditions in 131 women and 71 of their partners^12^. In China, Chan et al. conducted an ECS panel with 104 AR and X-linked conditions in 123 pregnant and 20 of their partners and reported 58.7% (n = 84) of them being carriers for at least one recessive condition^13^. Recently, Chetruengchai et al. analyzed 114 recessive genes in 1642 individuals in Thailand and identified the carrier frequency for at least one AR disorder, which was 39% (640/1642)^14^. Obviously, the more AR gene mutations are accounted for, the more carriers will be detected^1^.

63.6% of Vietnamese women carried at least one AR variant, which is quite prevalent. The finding has illustrated the actual context of high-risk carriers for AR and X-linked disorders that need to bring more actions to improve the quality of reproductive healthcare in Vietnam. Therefore, the establishment of a preconception and prenatal carrier screening panel is a righteous choice since it could detect Vietnamese-specific AR and X-linked conditions at an affordable cost.

### The most popular AR carriers

Understanding carrier frequency in the Southeast Asian population is still unclear due to limited data from genetic carrier screening in these countries. According to Lazarin et al., the carrier frequency in East Asians was generally lower than in Ashkenazi Jews and Caucasians (8.5% versus 43.6% and 32.6%)^15^. He also highlighted that *GJB2*-related hearing loss was the most common AR disorder (1 in 22), followed by beta-thalassemia disease (1 in 78) and SMA (1 in 85)^15^.

In the present study in Vietnam, 215 women were detected to carry at least one AR condition associated with 165 pathogenic and 124 likely pathogenic variants. These were similar to those of Chetruengchai et al. in Thailand (640 individuals carried 172 pathogenic and probably pathogenic variants)^14^. Considering the AR and X-linked condition with a frequency greater than 1.7% (1 in 60) as the most common disorders, we obtained seven top-carried genes in Vietnamese women, illustrated in Table 1. Generally, we could divide the common AR and X-linked conditions into 4 groups to discuss: the group of hearing impairment contained *GJB2* and *USH2A*; the group of genetic anemia including *HBB* and *G6PD* genes; the group of cystic fibrosis (*CFTR*); the group of metabolism disorders coded by *ATP7B* and *GAA* gene.

With higher rates of the most common recessive genes and a larger spectrum of AR and X-linked disorders, our results differed significantly from findings in Thailand and China. According to Chetruengchai et al., the top gene carrier frequency was *HBB* (19.6%), followed by *G6PD* (7.7%), then *HBA2* (3.96%)^14^. Besides, Shi et al. also indicated the first two common recessive genes were *HBA1/HBA2* and *HBB* (7.76% and 2.25%, respectively), and the next two common were *PAH* for phenylketonuria and *SLC26A4* for autosomal recessive deafness 4 (2.18% and 2.03%)^6^. Even when these three studies were conducted in the same Southeast and East Asian region, the AR and X-linked carriers presented differently due to different distributions of ethnicity, choices of screening methods, and the cutoff settings for the screening program. This would affirm the importance of building a Vietnamese-specific panel in the carrier screening program.

Compared to another study in Vietnam, our result was consistent with those reported by Tran et al., who also identified the highest recessive gene carrier frequency was *GJB2* (1 in 6), and the third common was *HBB* (1 in 23)^11^. Both studies provided strong evidence to take genes *GJB2* and *HBB* into a Vietnamese-specific carrier screening panel to detect early hearing impairment and inherited anemia in the preconception and prenatal care program.

Regarding other AR conditions recommended by Guo and Gregg in South and East Asia, and ACMG such as autoimmune polyendocrinopathy syndrome type 1, Usher’s syndrome type IIa, SMA, and fragile X syndrome^4,16,17^, those were not found or found 1% or below (1 in 100), so we did not take into our discussion.

### Hearing loss disorder (*GJB2* and *USH2A*)

Focused on the *GJB2*-related hearing loss gene, the c.109G>A (p.Val37Ile) variant was determined as the most prevalent pathogenic variant (85.1%) in Vietnam. This result was distinguished from those of Wattanasirichaigoon et al. in Thai reported p.V37I variant at 11.1%^18^, and Tsukada et al. in Japan identified c.235delC (p.L79fs) variant at 49.8%^19^. Each ethnic individual indeed provided specific pathogenic variants. The diversity of variant data obtained from different studies in South and East Asia has supported the design of an ethnic-based carrier screening program and enriched the landscape of pathogenic gene panels worldwide. We can confidently add *GJB2* with c.109G>A (p.Val37Ile) variant to the Vietnamese-specific ECS panel.

In literature, p.Val37Ile was often associated with bilateral mild to moderate hearing loss and adult-onset phenotype^20^. Nevertheless, it should be more cautious to explain to carrier individuals about their risks in preconception and prenatal counseling. In our study, among 57 women carrying pathogenic c.109G>A, four of them were found to be homozygotes and immediately underwent post-test counseling to perform auditory testing. An otolaryngologist confirmed their diagnosis as mild hearing loss, and they received a detailed follow-up auditory check for themselves and their offspring. Identifying *GJB2*-related hearing loss enabled women in our cohort to be aware of their status, either carried or suffered from chronic disability, and realize their risk of having offspring with AR conditions.

Besides *GJB2*-related hearing loss, we also identified 3.3% (1 in 31) of women carrying the *USH2A* gene that caused Usher syndrome, characterized by moderate to severe deafness at birth and progressive retinitis pigmentation^21^. The carrier frequency of Usher’s syndrome was determined at around 1 in 70 in most ethnic populations, according to Koenekoop et al.^22^. However, *USH2A* and Usher syndrome have not been documented in Vietnam. A *USH2A* carrier frequency of 1 in 31 in our study was even two and half times higher than the previous study that highlighted Vietnamese women at high risk of hearing impairment and vision issues. That’s why bringing *GJB2* and *USH2A* genes into the ECS panel in Vietnam is reasonable.

### Genetic anemia (*HBB* and *G6PD* deficiency)

Beta-thalassemia, a genetic anemia leading to life-threatening outcomes, was the second most common AR disorder (6%, 1 in 16) in the present study. Our report was quite consistent with those identified by Tran et al. in 2021 in Vietnam (1 in 23)^11^ but was distinguished from Shi et al., who found the most common AR condition was alpha-thalassemia with 7.8% (n = 103) through conducting a “small-size” ECS panel containing 11 AR and X-linked disorders in 1321 Chinese pregnant women^6^. This difference emphasized the ethnic-specific genes coded AR diseases and research participants.

Since the national program of genetic anemia screening has been widely implemented in Vietnam, what we obtained in this cohort provided positive support for actual target carrier screening for hereditary anemia in preconception and prenatal care. Indeed, the national genetic anemia carrier screening program not only enhanced the awareness and knowledge of this reduced life span condition but also detected 61.85% of fetuses carried alpha-thalassemia, 13.07% carried beta-thalassemia, and 0.61% carried others (HbE disease, alpha-thalassemia/HbE, and beta-thalassemia/HbE)^23^. Following this target carrier screening program, newborns can get an immediately detailed treatment and improve their quality of life.

G6PD deficiency, an inherited X-linked disorder, is known as a significant risk factor for hemolytic anemia in many Southeast Asians. In Thailand, Sathupak et al. found its prevalence was 19.8% (127/640)^24^, and Chetruengchai et al. determined the carrier rate of the *G6PD* gene was the third common at 3.4% (55/1642)^14^. Realizing early-onset anemia in newborns and its consequences, the newborn G6PD screening program has been conducted in several maternity centers since 2010 in Vietnam. Nevertheless, this postnatal screening program did not demonstrate a remarkable impact to improve genetic hemolytic anemia. The frequency of G6PD deficiency was reported to still be high in southern Vietnam (8.7% in ethnic Kinh and 14% in ethnic S’tieng), with the most common variant called *G6PD Viangchan*^25^. The *G6PD* gene carrier frequency in the present study was 3.6% (1 in 28), even lower than those reported by Hue et al.^25^, demonstrating the Vietnamese population is at high risk of hemolytic anemia. G6PD should be added to the Vietnamese-specific ECS panel to provide early diagnosis and treatment for this X-linked disorder in reproductive women and their partners.

### Cystic fibrosis

Cystic fibrosis (CF), an inherited disorder causing persistent lung infections and pancreatic insufficiency, has been seen as more common in individuals of Ashkenazi Jewish and Caucasian than other ethnicities, such as the African and Asian populations^4^. Since this condition is carried throughout individuals’ life spans, ACOG and ACMG recommended screening the CF gene in all women considering pregnancy^4,16^.

Unlike thalassemia awareness, the CF condition and its data had not been well documented in Vietnam. Additionally, no CF carrier screening is conducted in preconception and prenatal care. Herein, we had the CF carrier frequency (4.4%, 1 in 23), which kept the third common prevalence in the present study, and that was the first CF carrier screening report in Vietnamese women of reproductive age.

1 in 23 carried *CFTR* showed significantly close to the Ashkenazi Jewish (1 in 24) and Non-Hispanic White (1 in 25), considered a high-risk group of CF carriers. We wondered if the Vietnamese population is also a high-risk carrier of this AR disease. Recently, Vaidyanathan S et al. suggested that CF condition has become more common in South Asians with specific *CFTR* variants distinguished from non-Hispanic Whites^26^. Indeed, c.1210-11T>G pathogenic variant found 80% (12/15) can be determined as a specific *CFTR* variant of CF carriers in Vietnam. Regarding preconception and prenatal counseling, CF conditions can be effectively prevented if the affected individuals, including carriers and their offspring, are identified and intervened early. Two pregnant women at early gestational age (< 7 weeks) carried *CFTR* in our study. Both received post-test counseling to understand the risk of their child and scheduled a detailed scanning at 18 weeks of gestation to evaluate fetal soft markers to diagnose the possibility of postnatal CF early. Our finding facilitated the broadening of the genetic landscape of *CFTR* and added this gene to the Vietnamese-specific ECS panel.

### Metabolism disorders (*ATP7B* and *GAA*)

Interestingly, we also discovered two more common genes related to AR metabolism disorders that had not been mentioned in Vietnam yet. The first AR metabolism disease detected is Wilson’s disease, a disorder of copper metabolism coded by the *ATP7B* gene. Wilson’s disease has been considered the most common AR condition in Korea, with the carrier frequency found to be 1 in 53^27^, but its prevalence was nearly two times lower than our result (1 in 31, 3.3%). Recently, Yamaguchi et al. found the carrier frequency of this condition in Japan was 2.2%- 2.8%, which was still lower than our findings^28^. Wilson’s disease was one of the most common recessive conditions in Vietnam, and it needed to bring this gene was found in the Vietnamese ECS panel.

The second AR metabolism condition is glycogen storage disease type II, also called Pompe disease, another AR disorder caused by deficiency of the lysosomal alpha-glucosidase. This condition is characterized by an accumulation of glycogen within the lysosomes, causing progressive destruction of cardiac and skeletal muscle^29^. The sooner the diagnosis of this condition is made, the more the newborn could benefit from earlier enzyme replacement treatment^30^. Even though Pompe disease is found to be the most common disease in East Asia (1 in 77, 1.3%)^30^, this data has not been reported in Vietnam. In our cohort, *GAA* carrier frequency was found to be 1 in 56 (1.8%), which was one and a half times higher than the overall population, according to Park’s study^30^. It affirmed the feasibility of building an ECS panel with the *GAA* gene.

## Conclusions

In summary, genetic carrier testing has not been familiar to Vietnamese women in preconception and prenatal counseling because of the high-cost expense and unawareness of popular AR and X-linked diseases. Hence, our obtained results are considered the first essential foundation to confirm the benefit of genetic carrier screening and better understand the Vietnamese recessive genetic landscape. Based on current research, a Vietnamese-specific ECS panel for the seven most common recessive conditions was built from *GJB2, USH2A, HBB, G6PD, CFTR, ATP7B*, and *GAA*. This Vietnamese-specific ECS panel has responded to the actual clinical needs, matched with cost-effectiveness, and facilitated the provision of better genetic counseling in preconception and prenatal care in Vietnam.

## Methods

### Study methods and population

This cross-sectional study was conducted from December 1st, 2020, to June 30th, 2023. We collected data from non-pregnant women and pregnant women from 18 to 40 years old when they came to the Department of Obstetrics and Gynecology, University Medical Center–Branch 2 in Ho Chi Minh City. This study was conducted in a single medical center, University Medical Center–Branch 2, in Ho Chi Minh City. A total of 338 women were recruited in this study after giving their consent to participate.

3 obstetricians performed a pre-test counseling to collect personal information and explain the research procedure and benefits of ECS for 540 genes by using next-generation sequencing. Next, all women were taken a sample of 5 mL of blood in the peripheral vein kept in an EDTA tube. Then, these research samples were sent to the Molecular Biology Center to analyze 540 genes. Tran et al. reported a high prevalence of AR carriers in the Vietnamese population^11^. Hence, the cut-off of carrier frequency was defined as 1.7% (1 in 60) or greater, following the top popular list of AR gene mutations detected in this study. All recessive disorders with a prevalence greater than 1 in 60 are considered common diseases, and their genes will be added to the Vietnamese-specific carrier screening panel.

All results were given back to participants via both postal mail and email. Women detected carrying at least one recessive disease received post-testing counseling from 3 obstetricians and 1 genetic expert. Their partners were also invited to have ECS for 540 genes to predict the possibility of suffering from AR conditions in their children.

### ECS panel design for 540 recessive genes

Since the conception of carrier screening and the data of most common AR and X-linked conditions are very new and still unknown in Vietnam, we conducted a survey to collect the list of AR and X-linked disorders in 104 Vietnamese clinical experts, including obstetricians, pediatricians, neurologists, endocrinologist, geneticist, and family doctors in 7 specialized hospitals at Ho Chi Minh city. Based on the 113 American College of Medical Genetics and Genomics ACMG-recommended genes^16^ and suggestions from 104 Vietnamese clinical experts, 540 genes were accounted for in our cohort to evaluate the most common AR and X-linked disorders. Those genes were divided into 3 main groups based on the detrimental effect on quality of life in the population^4,16,31^. Group 1 presented life-threatening disorders, group 2 combined chronic conditions causing cognitive and physical impairment^31,32^, and group 3 had only 3 popular AR genes in Southeast Asia that included *GJB2, HBB*, and *G6PD*^14,17^.

### Targeted next-generation sequencing and bioinformatics data processing

DNA was extracted from peripheral blood using the QIAamp DNA Blood Mini Kit (Qiagen). Target enrichment was performed with the xGen Custom Hyb Panels (Integrated DNA Technologies, USA). Captured libraries were sequenced with 2 × 150 bp reads on the Nextseq/Novaseq platform (Illumina). Sequence reads were mapped onto the human reference genome hg38 using the BWA tool^33^. The Genome Analysis ToolKit was used for calling variants (SNPs and indels)^33^. Identified variants were annotated using ANNOVAR^34^. The average coverage depth was about 200×, with over 99% of the target regions covered by at least 20 reads.

### Variant interpretation

Variants with at least 20× coverage were analyzed using Geneyx (Geneyx Genomex, Israel)^35^ and interpreted according to recent ACMG guidelines^36^. Synonymous variants, intronic variants outside of the flanking regions, and variants with a minor allelic frequency (MAF) ≥ 5% in the Genome Aggregation (gnomAD) databases were excluded^37^. Using the Geneyx analysis platform, various in silico prediction programs, including SIFT, PolyPhen-2, MutationTaster, LRT, CADD, and REVEL, were used to analyze missense variants. The analysis of splicing effects was performed with SpliceAI and dbscSNV version 1.1 by AdaBoost and Random Forest, and GERP++ and PhyloP were used to explore nucleotide-specific estimates of evolutionary constraint. All detected pathogenic (P) and likely pathogenic (LP) variants were confirmed by Sanger sequencing. Carriers harboring P/LP variants were classified as genotype-positive. Non-carriers of P/LP variants were considered genotype negative.

### Statistics

Continuous variables were expressed as mean ± SD and non-parametric as median (interquartile range). Categorical variables were depicted using numbers (proportions).

### Ethics approval and consent to participate

The research protocol was approved by the Ethics Committee of the University of Medicine and Pharmacy at Ho Chi Minh City (Reference number: 369/HĐĐĐ-ĐHYD on June 2nd, 2020). All methods were performed in accordance with relevant guidelines and regulations.

## Data Availability

The data supporting this study’s findings are available from the Ho Chi Minh Department of Technologies and Sciences restrictions, but there are restrictions apply to the availability of these data, which were used under license for the current study and are not publicly available Data are, however, available from the authors upon reasonable request and with permission of the Ho Chi Minh Department of Technologies and Sciences. The detailed data and materials are available on request from Tuan-Anh Nguyen (anh.nt@umc.edu.vn) in accordance with current relevant policies and regulations.
